# Salvage robotic SBRT for local prostate cancer recurrence after radiotherapy: preliminary results of the Oscar Lambret Center

**DOI:** 10.1186/s13014-017-0833-9

**Published:** 2017-06-09

**Authors:** Thomas Leroy, Thomas Lacornerie, Emilie Bogart, Philippe Nickers, Eric Lartigau, David Pasquier

**Affiliations:** 1 Department of Radiation Oncology, Centre de Lutte Contre le Cancer Oscar Lambret, 3 rue Frédéric Combemale, 59020 Lille cedex, France; 2 Department of Radiation Physics, Centre de Lutte Contre le Cancer Oscar Lambret, 3 rue Frédéric Combemale, 59020 Lille cedex, France; 3 Department of Statistic, Centre de Lutte Contre le Cancer Oscar Lambret, 3 rue Frédéric Combemale, 59020 Lille cedex, France; 4 Université de Lille-CRIStAL UMR 9189, 59650 Villeneuve d’Ascq, France

**Keywords:** Stereotactic radiation body therapy, Prostate cancer, Recurrence

## Abstract

**Background:**

Currently, there is no standard option for local salvage treatment for local prostate cancer recurrence after radiotherapy. Our objective was to investigate the feasibility and efficiency of Robotic Stereotactic Body Radiation Therapy (SBRT) in this clinical setting.

**Methods/materials:**

We retrospectively reviewed patients who were treated at our institution with SBRT for local prostate cancer recurrence after External Beam Radiation Therapy (EBRT) or brachytherapy.

Multidisciplinary staff approved the treatment, and recurrence was biopsy-proven when feasible. A dose of 36 Gy was prescribed in six fractions. Treatment was delivered every other day.

**Results:**

Between August 2011 and February 2014, 23 patients were treated with SBRT for intra-prostate cancer recurrence with a median follow up of 22 months (6 to 40).

Twenty patients had biopsy-proven recurrence.

For 19 patients, EBRT was the initial treatment and in four patients, brachytherapy was the initial treatment; the median relapse-time from initial treatment was 65 months (28 to 150).

At relapse, 10 patients had an extra-capsular extension.

Fourteen patients were treated with androgen deprivation that could be stopped after a median of 1 month after SBRT (range 0–24).

A PSA decrease occurred in 82.6% of the patients after SBRT.

The 2-year disease-free survival and overall survival rates were 54 and 100%, respectively.

Disease progression was observed for nine patients (39.1%) (five local, three metastatic and one nodal progression) after a median of 20 months (7–40 months).

The median nadir PSA was 0.35 ng/ml and was achieved after a median of 8 months (1 to 30) after treatment.

We observed no grade 4 or 5 toxicity. Two patients presented with grade 3 toxicities (two Cystitis and one neuralgia). Other toxicities included urinary toxicities (five grade 2 and nine grade 1) and rectal toxicities (two grade 2 and two grade 1).

**Conclusion:**

SBRT for local prostate cancer recurrence seems feasible and well tolerated with a short follow up. Prospective evaluation is needed.

**Electronic supplementary material:**

The online version of this article (doi:10.1186/s13014-017-0833-9) contains supplementary material, which is available to authorized users.

## Introduction

Prostate cancer remains the most common cancer among men in developed countries. Worldwide, GLOBOCAN 2012 estimates 1,111,700 new cases in 2012 [1]. Radiotherapy plays a key role in the treatment of localized prostate cancer. However, recurrence can occur up to 25% in high-risk localized prostate cancer after external beam radiotherapy or brachytherapy. More than half of these recurrences are local recurrences [2]. Currently, there is no standard salvage treatment option for these recurrences, and early initiation of salvage androgen deprivation therapy (ADT) has not been proven to enhance survival [3, 4].

Several local treatment options have been tested. Post radiation prostatectomy is feasible with regard to high incontinence rates [5–8]. Other options such as brachytherapy, cryotherapy or high-intensity ultrasound ablation are currently investigated with some success in terms of efficiency and toxicity, but these options are limited by their availability and operator dependence [9–13]. Thus, local treatment is offered to less than 2% of the patients with a local recurrence [14]. Stereotactic radiation therapy (SBRT) is currently under development for primary prostate cancer treatment and shows excellent local control and manageable toxicities [15–18]. SBRT allows delivery of a high dose per fraction with a high shaped dose gradient. Higher dose fractions are especially interesting in prostate cancer with regard to the supposed low alpha/beta ratio of prostate cancer enabling a shortened overall treatment time [19]. Many radiotherapy centers are currently using SBRT or are implementing it because of its new indications in lung and brain tumors.

CyberKnife was installed in our institution in 2007 and has been used since 2011 to treat prostate cancer local relapses that are not eligible for other treatment modalities. Currently, few data are available on this treatment strategy. In this report, we retrospectively report the efficacy and the tolerance of SBRT in the reirradiation of prostate cancer in our institution.

## Materials and methods

From August 2011 to February 2014, 23 patients were treated with SBRT at our institution for recurrent prostate cancer after initial radiotherapy or brachytherapy. Initial characteristics of patients who are included in this study are described in Table 1.

**Table 1:** Patients characteristics

Median age at relapse (range)	70 (58-82)
Initial disease	
T-Stage	T1: 7 (30.4%) T2: 8 (34.8%) T3: 7 (30.4%) NA: 1 (4.3%)
Median PSA	10.38 ng/mL (2.34–57)
Gleason	5: 1 (4.3%). 6: 5 (21.7%). 7: 13 (56.5%). 9 :1 (4.3%) NA: 3 (13%)
Prior RT modality	
External beam radiotherapy	19 (83%)
Brachytherapy	4 (17%)
Median dose of EBRT (Gy) (range)	75.6 (70–75.6)
Residual treatment urinary or rectal toxicities	Yes : 11 (Grade 1 : 7. Grade 2 : 4) No = 12
Median interval from prior RT to salvage (range)	65 months (28 to 150)
Relapse	
Biopsy at relapse	Yes: 19 (83%). No: 4 (17%)
Unilateral positive biopsies	9 (47%)
Bilateral positive biopies	10 (53%)
Number of positive biopsies : median (range)	4 (1–13)
MRI	Yes: 23 (100%). No 0 (0%)
Involvment on MRI	Unilateral: 12 (52.2%) Bilateral: 10 (43%) None: 1 (4%)
Extracapsular invasion on MRI	11 (47%)
Choline PET scan	Yes: 21 (91.3%) No: 2 (8.7%)
Androgen deprivation therapy	Yes: 14 (61%) No: 9 (39.1%)

Recurrence diagnoses were based on Phoenix criteria and were biopsy-proven when feasible. Recurrence must occurred at least 2 years after the initial treatment. Initial treatment was EBRT for 19 patients and brachytherapy for four patients; these patients had a median relapse time of 65 months (28 to 150) from initial treatment. Fourteen patients were treated with androgen deprivation; of them, four patients had androgen deprivation prior to SBRT for biochemical relapse.

All patients had an MRI and 21 had a choline PET.

On MRI, 10 patients had bilateral relapses, and 10 had an extra-capsular invasion. SBRT treatment had to be decided by multidisciplinary staff and when no other local option, such as high-intensity ultrasound, was feasible or available.

This retrospective, single-institution, cross-sectional study was approved by our Institutional Committee on Human Research.

### Treatment planning and delivery

All patients were treated with CyberKnife. Fiducials were implanted before treatment. Delineation was made on a planning-CT that was registered with the pre-treatment MRI when the fiducials were visible on MRI.

Depending on pre-treatment data, such as MRI or biopsy results, the planning target volume (PTV) could be obtained from a 2 mm margin applied to the Clinical Tumor Volume (CTV) that was defined as the entire prostate or the half-prostate or applied to the Gross Tumor Volume (GTV) that was defined as the recurrence observed on multiparametric MRI (T1 and T2-weightened, diffusion and perfusion sequences). MRI sequence used to delineate the GTV was chosen by a radiologist and was the sequence in which the tumor was the most visible.

A total dose of 36 Gy was prescribed to the 80% isodose line (95% PTV coverage) in six fractions of six Gy. The radiation oncologist decided to give priority to PTV or the organ-at-risk depending on the clinical context. Treatment was delivered every other day with CyberKnife during 12–14 days, and real-time tracking of the intra-fraction was used.

Normal tissue constraints used for planning are summarized in Table 2.

**Table 2:** Dose constraints

Organ-at-risk	Dose constraint
Rectum	V27 < 2 cc
	V12 < 20%
Bladder	V27 < 5 cc
	V12 < 15%
Intra-prostatic urethra	V24 < 30%
	V36 < 1 cc

### End points

Disease-free survival was defined as the time between the first treatment session and the occurrence of a biochemical recurrence. The Phoenix criteria were used to define a biochemical recurrence. Local disease-free survival was defined as the time between the first treatment session and the occurrence of a local recurrence that was diagnosed by positive biopsy after treatment. Toxicities were assessed using the Common Terminology Criteria for Adverse Events, version 4.0.

### Statistics

Stata v11.2 (StataCorp. 2009. Stata Statistical Software: Release 11. College Station, TX: StataCorp LP) was used for the statistical analyses.

Time to distant or local recurrence was defined from the first treatment session. Rates were estimated using the Kaplan-Meier method. The univariate analyses of local control were performed using the Cox regression model. A *p* value <0.05 was chosen as the significance threshold.

## Results

### Treatment

Treatment was delivered in six fractions over a median of 15 days. Median follow-up was 22.6 months (range 6 to 40 months). Median PSA pre-SBRT was 2.5 ng/ml (0–11.7). Nineteen patients had whole-gland treatment, three had focal treatment, and one had hemi-prostate treatment. Androgen deprivation was stopped after a median of 1 month after SBRT (range: 0–24).

#### Efficacy

After treatment, a PSA decrease occurred in 19/23 patients. Median nadir PSA was 0.35 ng/ml and was achieved after a median of 8 months (1 to 30) after treatment.

**Fig. 1:**
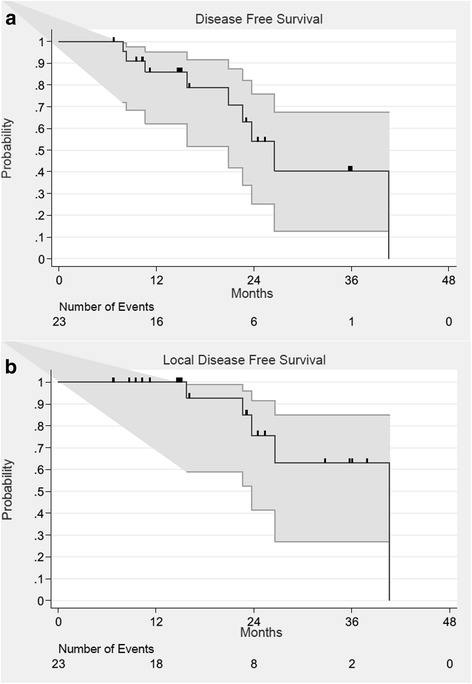
Disease-Free (**a**) and Local disease-free (**b**) survival curves. *Grey Lines* represent 95% confidence intervals

Disease progression was observed for nine patients (39.1%) after a median of 20 months (7–40 months). The 2-year disease-free survival and overall survival were 54 and 100%, respectively. Median DFS was 27 months (IC95% 20.9-… months). Five local relapses were observed. One and 2-year local disease free survival were 100 and 76%, respectively (Fig. 1). We performed a univariate analysis to identify prognostic factors affecting local control, but no factor was found especially pre-ADT treatment or MRI extracapsular extension (Additional files 1 and 2: Figures S1 and S2).

Dosimetric results are detailed in Table 3.

**Table 3:** Dosimetric results

Dosimetric results	Median (range)
Whole-prostate Treatment	*n* = 19
PTV volume (cm3)	48 (16–131)
PTV D98 (Gy)	30.4 (8.7–36)
PTV D95 (Gy)	32.5 (13–36.5)
PTV D50 (Gy)	38.3 (35.2–40.2)
PTV D2 (Gy)	42.9 (39.3–46.9)
CTV volume (cm3)	29.5 (6–87.2)
CTV D98 (Gy)	35.8 (11.1–37,7)
CTV D95 (Gy)	36.3 (15.6–38.3)
CTV D50 (Gy)	38.8 (36.2–41)
CTV D2 (Gy)	42.2 (39.38–47.27)
Hemi-prostate Treatment	*n* = 1
PTV volume (cm3)	34.1
PTV D98 (Gy)	14.83
PTV D95 (Gy)	22.5
PTV D50 (Gy)	39.67
PTV D2 (Gy)	43.79
CTV volume (cm3)	22.8
CTV D98 (Gy)	21
CTV D95 (Gy)	27.78
CTV D50 (Gy)	40.41
CTV D2 (Gy)	43.97
Focal treatment	*n* = 3
GTV volume (cm3)	3.7 (0.6–6.9)
GTV D98 (Gy)	37.6 (37.58–37.71)
GTV D95 (Gy)	38.1 (37.99–38.16)
GTV D50 (Gy)	39.77 (39.32–40.23)
GTV D2 (Gy)	41.2 (41.19–41.22)
Organ-at-risk	*n* = 23
Dmax rectum (Gy)	34.8 (6.8–42.26)
D2cc rectum	23.83 (4–36.3)
V12 rectum (cm3)	8.2 (0–44.2)
V27 rectum (cm3)	1.3 (0–14.7)
Dmax bladder (Gy)	36.2 (15.9–41.9)
D5cc bladder (Gy)	25.3 (6.7–37.4)
V12 bladder (cm3)	16 (0.2–108)
V27 bladder (cm3)	3.2 (0–26.8)

### Toxicities

Overall, the treatments were well tolerated. We observed no grade 4 or 5 toxicity. Two patients presented with grade 3 toxicities (two cystitis and one neuralgia). Other toxicities included urinary toxicities (five grade 2 and nine grade 1) and rectal toxicities (two grade 2 and two grade 1); these are described in Table 4.

**Table 4:** Toxicities according NCI-CTCAE V4

Toxicities	n (%)
Grade 1	13 (56.5%)
Cystitis	9 (41%)
Dysuria	1 (4.3%)
Urethritis	1 (4.3%)
Proctitis	2 (8.7%)
Grade 2	9 (39.1%)
Dysuria	1 (4.3%)
Proctitis	2 (8.7%)
Cystitis	4 (17.4%)
Urethral stenosis	2 (8.7%)
Grade 3	3 (13%)
Cystitis	2 (8.7%)
Neuritis	1 (4.3%)

We found no correlation between dosimetric factors, previous treatment toxicities or post-SBRT treatment.

We observed 2/4 (50%) and 10/19 (52%) grade 2 or higher toxicities after SBRT for brachytherapy and for EBRT failures respectively.

Grade 2 or higher toxicities was 11/19 (58%) and 0% after whole gland and less than whole gland SBRT respectively.

## Discussion

Currently, despite many options for intra-prostatic relapse after radiotherapy, there is no standard of care, leading to an underuse of local treatments.

Prostatectomy was the first treatment used, but the risk of complications is greater than the risk encountered in patients who have not received prior radiotherapy. In the largest published series, the specific 5-year survival rates were 79% [20] and 85% [21]. Survival without relapse was 43% at 10 years and 58% at 5 years [20, 21]. In a recent systemic review of the literature consisting of 404 patients, the non-relapse survival rates at 5 and 10 years varied from 47 to 82% and from 28 to 53%, respectively. The cancer-specific and overall survival rates at 10 years ranged from 70 to 83% and from 54 to 89%, respectively [22]. Compared with prostatectomy in patients who had not been previously treated, salvage prostatectomy after radiotherapy is burdened by significantly higher urinary and gastrointestinal morbidity. Gotto et al. [6] reported that the rates of urethral stenosis, urinary retention, urinary fistula and rectal injury were 47, 25, 4 and 9%, respectively. The functional outcomes were also significantly worse, with frequent urinary incontinence (21 to 90%) and impotence observed in nearly all patients. High-intensity focused ultrasound (HIFU) is a more recently developed treatment. The literature is dominated by a series published by a French team, with 290 patients treated between 1995 and 2009 [23]. The mean interval between radiotherapy and HIFU was 60 (±22) months. The mean pre-HIFU PSA level was 6.38 (±7.61) ng/ml. Half of the patients also received hormone therapy. Survival without relapse at 5 years following HIFU was 45, 31 and 21%, which was dependent on whether the patients belonged to the favorable-, intermediate- or high-risk D’Amico group, respectively, prior to their initial treatment. In this cohort, the grade 1, 2 and 3 urinary incontinence levels were 23, 14 and 9%, respectively. Nearly 8% of patients required an artificial sphincter following HIFU. Importantly, pubic osteitis occurred in 2.5% of patients despite adherence to parameters specific to HIFU following radiotherapy [23].

Cryotherapy is another recently described warm ablation treatment. The results of the few published studies were disappointing. In a retrospective multicenter series pooling 279 patients, survival without biochemical relapse at 5 years was 54%. Prostatic biopsies showed tumor persistence in 32% of patients following cryotherapy [24]. In a paired case-controlled study, prostatectomy and cryotherapy were compared following radiotherapy. Survival without relapse at 5 years was significantly lower after cryotherapy (21% vs. 61%, *p* < 0.05); this finding was also applied to the overall survival rate [25]. In a published retrospective series, the rates of incontinence, urinary retention, recto-urethral fistulae and impotence varied from 5 to 73%, 3 to 67%, 1 to 3% and 72 to 83%, respectively. Low- and high-dose rate brachytherapy has also been described as a salvage treatment following radiotherapy. Most of the available data stem from retrospective studies. In a recently published phase II study involving 42 enrolled patients, survival without biochemical relapse at 5 years following high-dose-rate brachytherapy was 69% with a median survival time of 36 months; 15% of the patients presented grade 2 toxicity, and one patient presented grade 3 incontinence [26]. Toxicity appears to be more marked in the older series, which registered 46% of cases at grade 2 and 11% of cases at grade 3 toxicity [27]. A phase II study of the Radiation Therapy Oncology Group (RTOG) has recently been completed, and the results are still being processed; a phase II study is currently being performed in France. Similar to the other salvage treatments, patients presenting with a differentiated tumor before external radiotherapy (Gleason score ≤ 7), a long recurrence-free interval, a PSA value below 10 ng/ml and a PSA doubling time greater than 10 or 12 months on relapse are most likely to benefit from salvage brachytherapy.

SBRT appears to be a promising option because of the high dose per fraction radiobiology and its ability to spare normal tissues. However, few series have currently reported the recurrence setting. Our study, despite being retrospective, showed that SBRT is effective and well tolerated in treating prostate cancer recurrence with a short follow-up.

The literature consists of small-sized series, making it difficult to assess and compare dose and fractionation. In our study, we used a dose of 36 Gy in six fractions; this regimen obtained good results in terms of efficacy. In a similar study, Jereczek-Fossa et al. treated 15 patients with robotic SBRT. At a median follow-up of 10 months, a PSA reduction superior to 50% was noted in only 11 of 15 patients, and one-third of their patients presented with early biochemical progression.

The dose used in our series is higher than that described by Jereczek-Fossa et al. (30 Gy in five fractions), which has been described as being too low [28, 29], and lower than that applied in the study by Fuller et al. [29], which was consistent with this team’s own practice (heterogeneous prescription similar to high-dose-rate brachytherapy).

*Fuller and al*., who used SBRT as an HDR-like technique, shows results that seem better in terms of disease-free-survival with a similar toxicity [29]. This could be explained by the HDR-like technique used and the higher dose (34 Gy in five fractions) delivered suggesting that higher doses are necessary to achieve local control. Nevertheless, 10 patients in our cohort had extra-capsular invasion on MRI, which could also explain the better DFS in the Fuller cohort.

Despite the fact that CyberKnife theoretically has an infra-millimetric precision on a phantom, we believe a PTV margin of 2–3 mm is warranted to take into account uncertainties such as prostate or fiducial motions and edema and to better reflect dose distribution. Dose constraints were empiric based on previous HDR brachytherapy and SBRT experience. To identify specific dose constraints, it is necessary to obtain the most realistic and reproducible dose distribution.

Nevertheless, further follow-up is needed to evaluate late toxicities. Moreover, the median dose previously delivered by EBRT was 73.8 Gy in the *Fuller* series and 75.6 Gy in ours, corresponding to a lower dose than that currently used for prostate cancer treatment. Increased toxicities may appear for patients previously treated by EBRT with a higher dose.

Short androgen deprivation therapy had been widely prescribed in our institution. Its role and its synergic action with SBRT are still unclear. However, it allows a reduction in prostate volume to obtain a better dose distribution.

Focal treatment is currently a hot topic in prostate cancer treatment. We observed two relapses in three patients who had focal SBRT. This is why we would recommend a mapping biopsy of the prostate before focal treatment.

Furthermore, biopsies showed bilateral extension for 46% of the patients who had only a unilateral positive multiparametric MRI ± 18 F-fluorocholine PET. Thus, Kanoun and al. shows that despite good sensibility of multiparametric MRI and 18 F-fluorocholine PET, prostate biopsy remains the gold standard [30].

Because of the small number of patients included in this study, no prognostic factor could be identified. Currently, no conclusions can be made on available data, but early results are promising. Prospective studies are warranted to identify which patients benefit from local treatment.

## Conclusion

SBRT for local prostate cancer recurrence seems feasible and well tolerated with short follow-up. Prospective evaluation with long follow up is needed before routine clinical use and this technique should be used with caution by teams with large experience in SBRT currently. SBRT dose prescription homogenization is required to start such trials. Further evaluation is needed to identify patients who would benefit most from this treatment.

## Additional files

Additional file 1: Figure S1. Disease-Free (A) and Local disease-free (B) according to extracapsular extension at MRI survival curves. (JPG 167 kb)

Additional file 2: Figure S2. Disease-Free (A) and Local disease-free (B) according to ADT treatment. (JPG 149 kb)

## Abbreviations

ADT: Androgen deprivation therapy
CTV: Clinical Tumor Volume
EBRT: External Beam Radiation Therapy
GTV: Gross Tumor Volume
HIFU: High-intensity focused ultrasound
PTV: Planning target volume
RTOG: Radiation Therapy Oncology Group
SBRT: Stereotactic Body Radiation Therapy
