# Supplementary figures and images for: Salvage robotic SBRT for local prostate cancer recurrence after radiotherapy: preliminary results of the Oscar Lambret Center
Source: Radiat Oncol. 2017 Jun 9;12:95. doi: 10.1186/s13014-017-0833-9 (PMC5466739; doi:10.1186/s13014-017-0833-9)

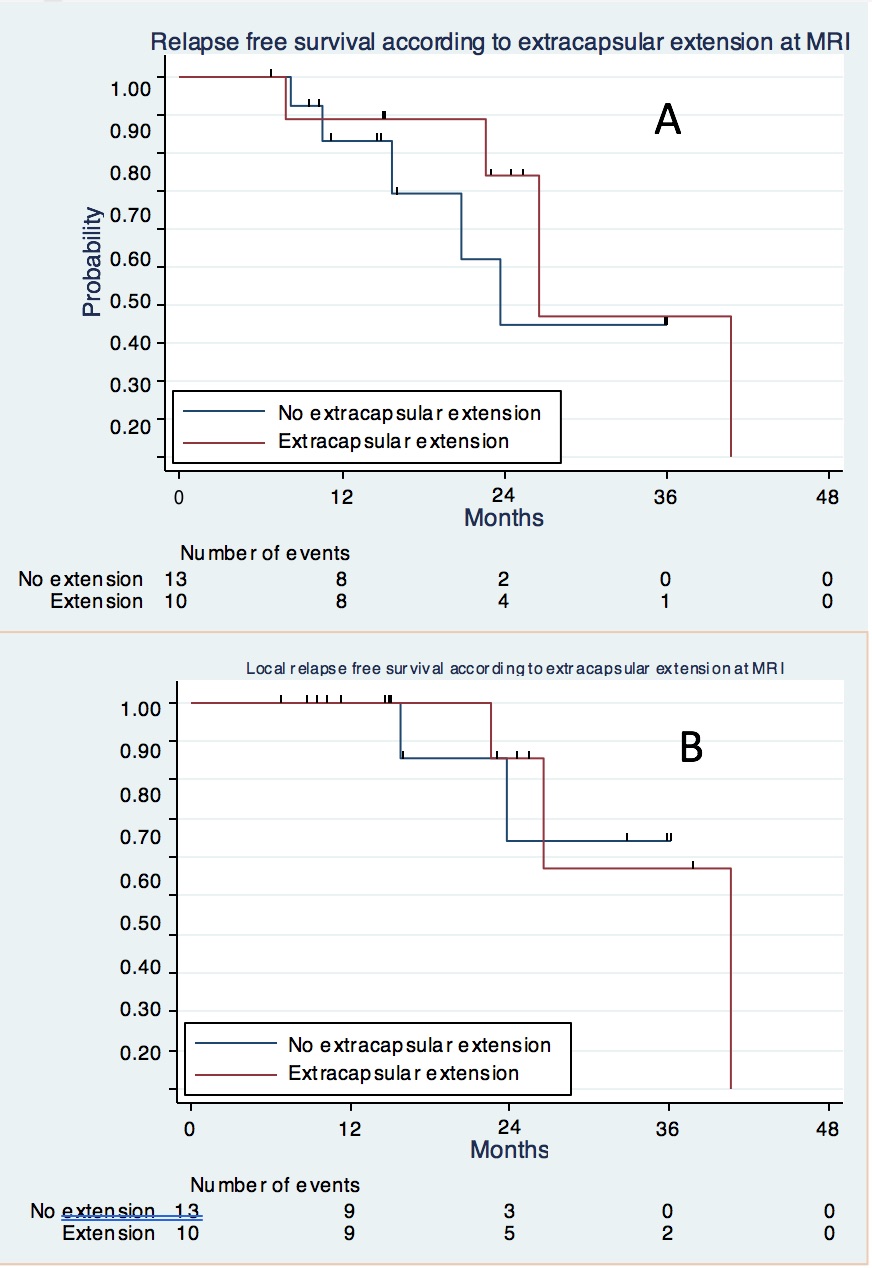

Supplement: Supplementary file 1 — Disease-Free (A) and Local disease-free (B) according to extracapsular extension at MRI survival curves. (JPG 167 kb) [file 13014_2017_833_MOESM1_ESM.jpg]

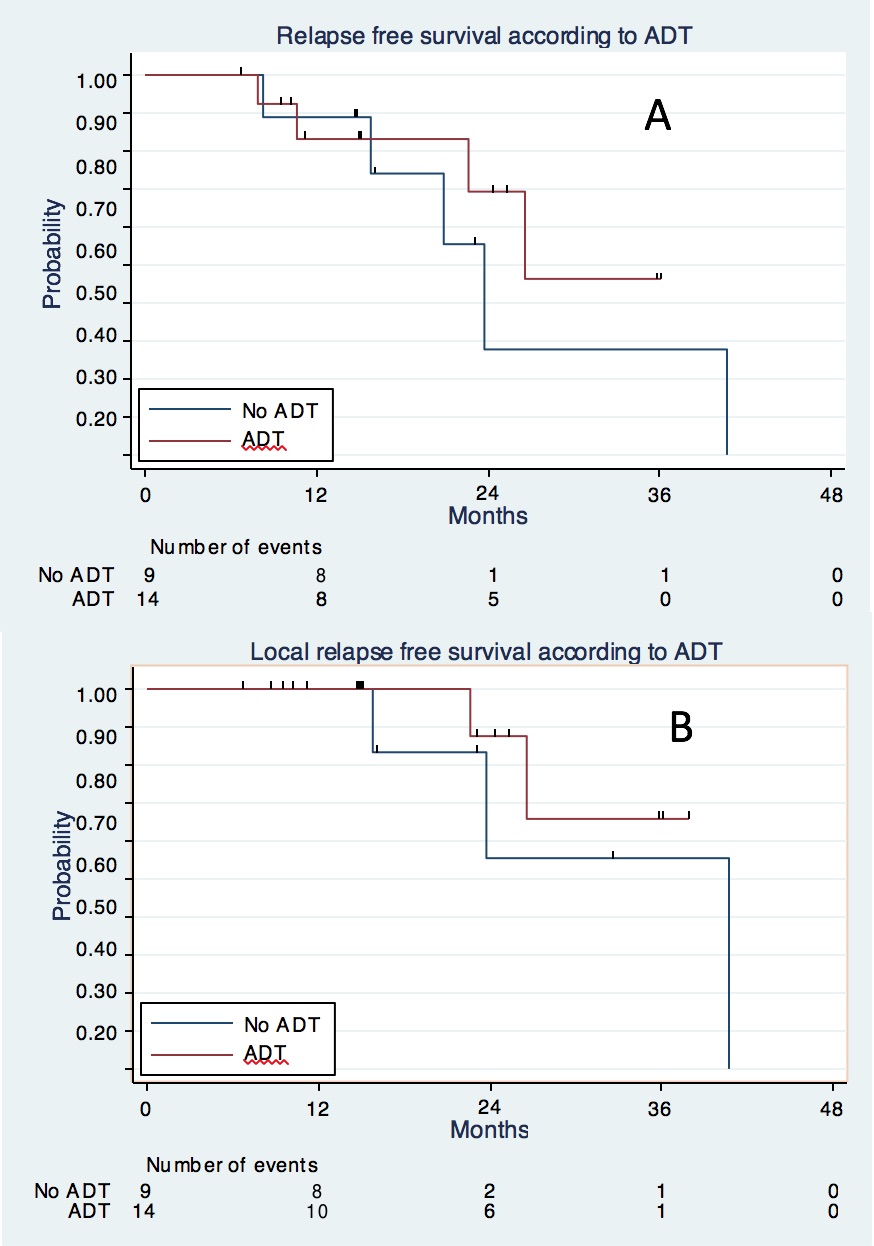

Supplement: Supplementary file 2 — Disease-Free (A) and Local disease-free (B) according to ADT treatment. (JPG 149 kb) [file 13014_2017_833_MOESM2_ESM.jpg]
